# Targeting the gut-immune-brain axis: pharmacological insights from depression in inflammatory bowel disease

**DOI:** 10.3389/fphar.2026.1793292

**Published:** 2026-04-01

**Authors:** Júlia Leão Batista Simões, Geórgia de Carvalho Braga, Charles Elias Assmann, Margarete Dulce Bagatini

**Affiliations:** 1 Graduate Program in Biochemistry, Federal University of Santa Catarina (UFSC), Florianópolis, Brazil; 2 Medical School, Federal University of Fronteira Sul, Chapecó, Brazil; 3 Graduate Program in Biological Sciences, Toxicological Biochemistry, Federal University of Santa Maria, Santa Maria, Brazil; 4 Graduate Program in Medical Sciences, Federal University of Fronteira Sul, Chapecó, Brazil

**Keywords:** depression, dysbiosis, gut-brain axis, inflammatory bowel disease, kynurenine pathway, psychobiotics

## Abstract

Inflammatory Bowel Disease (IBD), comprising Crohn’s Disease and Ulcerative Colitis, is a chronic inflammatory condition of the gastrointestinal tract with a remarkably high prevalence of psychiatric comorbidities, particularly Major Depressive Disorder (MDD). The traditional monoaminergic hypothesis of depression is insufficient to explain the complex etiology of MDD, paving the way for new paradigms, such as the inflammatory hypothesis of depression. This narrative review critically explores IBD as a human clinical model to investigate the connection between chronic inflammation and depression. It is argued that gut dysbiosis, a central feature of IBD, is a fundamental trigger that, through a compromised gut barrier, drives systemic inflammation and, subsequently, neuroinflammation. We detail the molecular and cellular mechanisms that link intestinal inflammation to central nervous system (CNS) dysfunction, including microglial activation, hypothalamic-pituitary-adrenal (HPA) axis dysregulation, and kynurenine pathway activation, which diverts tryptophan metabolism from serotonin synthesis to the production of neurotoxic metabolites. Robust epidemiological evidence demonstrating a bidirectional association between IBD and depression is discussed, suggesting a shared pathophysiology rather than a simple cause-and-effect relationship. Furthermore, we review the implications and emerging therapeutics, highlighting the antidepressant effects of immunobiologicals, such as anti-TNF therapies, and the potential of emerging interventions that target the microbiome, such as probiotics, psychobiotics, fecal microbiota transplantation, and anti-inflammatory diets. Furthermore, we address the limitations of the current literature, such as the lack of a quantitative definition for dysbiosis and the scarcity of clinical trials with integrated neuropsychiatric outcomes, and propose directions for future translational research. We conclude that IBD should be considered a systemic disease with significant psychiatric repercussions, advocating for an integrated therapeutic approach that combines immunomodulatory, neuromodulatory, and microbiological interventions to treat both gut and brain pathology effectively.

## Graphical Abstract

**Figure:**
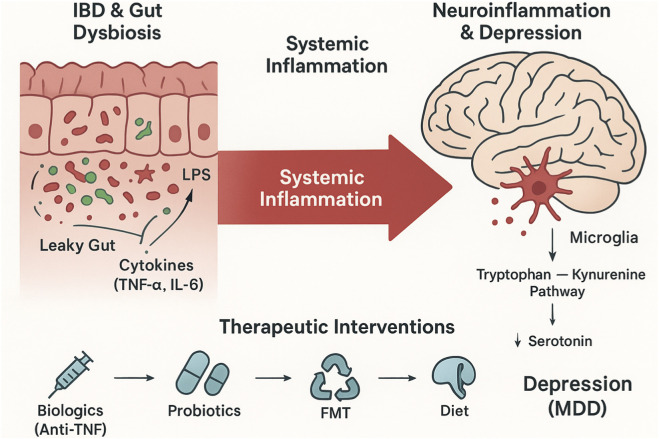

## Highlights

- Inflammatory Bowel Disease (IBD) and Major Depression (MDD) are strongly linked by shared inflammatory pathways.
- Dysbiosis and intestinal barrier failure in IBD drive systemic inflammation through the translocation of microbial components such as LPS.
- Systemic inflammation causes neuroinflammation by activating microglia and compromising the blood-brain barrier.
- Neuroinflammation diverts tryptophan metabolism from serotonin production to the neurotoxic kynurenine pathway.
- Anti-inflammatory therapies, such as biologics (anti-TNF), demonstrate antidepressant effects, supporting an integrated treatment approach.

## 1 Introduction

Inflammatory Bowel Disease (IBD), comprising Crohn’s Disease (CD) and Ulcerative Colitis (UC), is a chronic, immune-mediated disorder of the gastrointestinal tract that has reached the proportions of a global epidemic, with prevalence rates now exceeding 0.3% in Western nations and rising rapidly in newly industrialized regions (Wang et al., 2026). Current pharmacological management focuses on a “treat-to-target” strategy, utilizing a progression from aminosalicylates and corticosteroids to immunomodulators and highly targeted biological therapies, such as anti-TNF-α (infliximab), anti-IL-12/23 (ustekinumab), and anti-integrin (vedolizumab) agents (Stallmach et al., 2023). While these therapies have revolutionized the achievement of mucosal healing, significant limitations persist. Up to 40% of patients fail to respond or lose response to biologics, and many in clinical remission continue to suffer from debilitating extraintestinal manifestations (Abreu et al., 2020).

Among these, Major Depressive Disorder (MDD) is arguably the most pervasive and clinically significant comorbidity. Depression in IBD is not merely a psychological reaction to chronic illness; it is a biological driver of disease activity (Stallmach et al., 2023). Comorbid depression is associated with a two-to-three-fold increase in IBD flares, a higher risk of hospitalization and surgery, and reduced medication adherence (Hu et al., 2021). Furthermore, evidence suggests that depression may precede the clinical diagnosis of IBD by years, pointing toward shared early-life risk factors and a bidirectional pathophysiological link (Di Vincenzo et al., 2025). Integrating the management of depression into IBD treatment is, therefore, not an elective addition but a clinical necessity for improving long-term outcomes (Abdelbadiee et al., 2025). This narrative review explores the gut–immune–brain axis as the unifying pharmacological framework to address this unmet need.

The source of this chronic, low-grade inflammation is increasingly attributed to the gastrointestinal tract. The microbiota-gut-brain axis (MGBA) has emerged as a fundamental bidirectional communication network that connects intestinal health, microbial activity, and peripheral immunity with central nervous system (CNS) function, including mood and cognition (Appleton, 2018; Clapp et al., 2017). This complex axis uses multiple signaling pathways—neural, endocrine, immune, and metabolic—to maintain homeostasis. Dysfunction in this axis, particularly dysbiosis of the gut microbiota, is now recognized as a contributing factor to a variety of neurological and psychiatric disorders, including depression (Patel et al., 2025).

In this context IBD emerges as an invaluable human clinical model (Craig et al., 2022; Di Petrillo et al., 2025). IBD is characterized by chronic, relapsing-remitting, high-grade inflammation in the gastrointestinal tract, resulting from a dysregulated immune response to the gut microbiota in genetically susceptible individuals. The extremely high prevalence of depression in this population transcends the notion of a simple psychological reaction to the burden of a chronic illness (Fakhfouri et al., 2024). Consequently, IBD offers a unique opportunity to investigate, *in vivo*, how a well-defined peripheral inflammatory state impacts CNS function and behavior.

**FIGURE 1:**
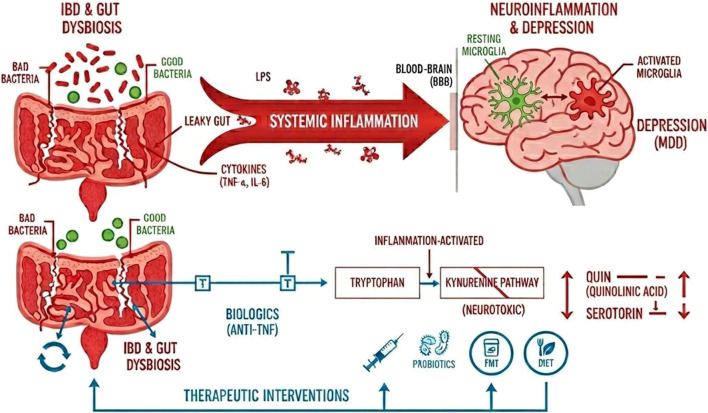
Impact of Gut Dysbiosis and Neuroinflammation on Depression via the Kynurenine Pathway. A proposed mechanism illustrating the role of the gut-brain axis in Major Depressive Disorder (MDD). The top panel shows how gut dysbiosis in Inflammatory Bowel Disease (IBD) leads to a “leaky gut” and the release of pro-inflammatory cytokines (e.g., TNF-α, IL-6) and LPS. This drives systemic inflammation, which crosses the blood-brain barrier (BBB) to activate microglia, resulting in neuroinflammation and depression. The bottom panel details the biochemical pathway, where inflammation shunts tryptophan metabolism away from serotonin production and towards the neurotoxic kynurenine pathway. Therapeutic interventions, such as biologics (anti-TNF), probiotics, Fecal Microbiota Transplant (FMT), and diet, aim to block this inflammatory cascade and mitigate depressive symptoms.

This narrative review will, therefore, critically examine the scientific literature supporting the thesis that gut dysbiosis associated with IBD is a key etiological factor in the pathogenesis of comorbid depression. The central argument is that IBD, through the propagation of systemic inflammation and subsequent neuroinflammation, acts as a biological trigger for the development of depressive symptoms. We propose that IBD should be understood not just as a disease of the gut, but as a systemic condition with profound neuropsychiatric consequences, which demands a paradigm shift toward an integrated and multifaceted therapeutic approach (Figure 1).

To maintain a high standard of academic rigor and transparency, the selection of literature for this narrative review was guided by a structured search strategy designed to capture high-impact, peer-reviewed evidence regarding the pharmacological and neuroimmunological intersections of IBD and MDD. Comprehensive literature searches were performed across multiple electronic databases, including PubMed/MEDLINE, Web of Science (WoSCC), Scopus, and the Cochrane Library. The primary timeframe focused on research published between 2010 and 2025, prioritizing the most recent meta-analyses, randomized controlled trials (RCTs), and landmark mechanistic studies, while retaining classic studies essential for the historical context of the inflammatory hypothesis.

Search terms included a combination of MeSH terms and keywords such as “Inflammatory Bowel Disease,” “Crohn’s Disease,” “Ulcerative Colitis,” “Depression,” “Major Depressive Disorder,” “Microbiota-Gut-Brain Axis,” “Neuroinflammation,” “Kynurenine Pathway,” “Tryptophan Metabolism,” “Probiotics,” “Psychobiotics,” and “TNF-alpha inhibitors.” Inclusion criteria were defined to select: (1) peer-reviewed original research and high-quality reviews; (2) studies specifically investigating the bidirectional relationship between intestinal inflammation and neuropsychiatric symptoms; (3) clinical trials evaluating the impact of immunomodulators or microbiome-targeted therapies on mental health outcomes; and (4) preclinical models elucidating molecular signaling pathways.^2^ Conversely, exclusion criteria were applied to non-English language publications, meeting abstracts without full texts, and studies lacking a clear mechanistic focus on the gut–brain–immune triad. This systematic approach to a narrative synthesis ensures that the pharmacological insights provided are grounded in the most robust and up-to-date data available as of 2025.

## 2 Gut microbiota and the gut-brain axis

The human gastrointestinal tract is colonized by a dense and diverse community of microorganisms—including bacteria, viruses, fungi, and archaea—collectively known as the gut microbiota (Evrensel and Ceylan, 2015). Far from being a passive commensal, this “microbial organ” establishes a symbiotic relationship with the host and performs vital functions for health. The gut microbiota is fundamental to host physiology. Its metabolic functions include the fermentation of non-digestible dietary fibers, resulting in the production of crucial metabolites like short-chain fatty acids (SCFAs), the synthesis of essential vitamins (e.g., vitamin K and B-complex), and the metabolism of bile acids and xenobiotics. Furthermore, the microbiota plays a protective role, competing with pathogens for nutrients and ecological niches and producing antimicrobial substances, forming a barrier against colonization by invasive microorganisms.

The initiation of intestinal immune imbalance in IBD is driven by a profound ecological shift away from microbial “tolerance.” Under healthy conditions, the microbiota maintains homeostasis through the production of short-chain fatty acids (SCFAs), particularly butyrate, which promote the differentiation of anti-inflammatory regulatory T cells (Treg) and suppress pro-inflammatory Th1 and Th17 cell maturation (Acevedo-Román et al., 2024). However, in IBD, a significant depletion of SCFA-producing genera (e.g., *Faecalibacterium*, *Roseburia*) leads to a loss of this “immunological brake” (Góralczyk-Bińkowska et al., 2022; Luo et al., 2025). This niche is often occupied by an expansion of *Proteobacteria* and *Enterobacteriaceae*, which exhibit high levels of Pathogen-Associated Molecular Patterns (PAMPs), such as lipopolysaccharide (LPS) and flagellin (Ciesielska et al., 2020; Yassin et al., 2025).

These microbial ligands bind to Toll-like receptors (TLRs), specifically TLR4, on the surface of intestinal dendritic cells and macrophages, triggering the nuclear factor kappa B (NF-κB) pathway. This results in the hypersecretion of IL-12 and IL-23, which further drives the differentiation of pathogenic Th17 cells (Ciesielska et al., 2020). These Th17 cells secrete IL-17 and TNF-α, which directly disrupt the expression of tight-junction proteins (e.g., zonula occludens, claudins), creating the “leaky gut” state that allows massive translocation of LPS into the systemic circulation (Ertenli et al., 2012; Hu et al., 2021). Thus, dysbiosis initiates immune imbalance through a two-hit mechanism: the loss of tolerogenic metabolites and the active stimulation of innate immunity by pathobionts, establishing the systemic inflammatory cascade that culminates in neuroinflammation (Abdelbadiee et al., 2025; Acevedo-Román et al., 2024).

Of particular relevance to this review, the gut microbiota is an indispensable modulator of the host’s immune system (Clemente-Suárez et al., 2024; Evrensel and Ceylan, 2015). Since birth, microbial colonization educates and shapes the development of both innate and adaptive immunity, both locally in the gut (gut-associated lymphoid tissue, or GALT) and systemically. The microbiota regulates the balance between T cell subpopulations, such as the pro-inflammatory T helper 17 (Th17) cells and the anti-inflammatory regulatory T cells (Treg), a crucial balance for maintaining immune tolerance.

Communication between the gut and the brain is bidirectional and occurs through multiple interconnected pathways, which constitute the MGBA. The gut microbiota is at the heart of this communication, functioning as a transducer that converts environmental signals (like diet and stress) into biological signals that the CNS can interpret. The main communication pathways are summarized in Table 1 and detailed below.

**TABLE 1:** Main communication pathways of the microbiota-gut-brain axis.

Communication route	Key mediators	Mechanism of action	Effect on the CNS/Behavior
Neural	Vagus nerve	Direct afferent signaling (gut-to-brain) via metabolites and cytokines to the brainstem (e.g., nucleus of the solitary tract) (Bonaz et al., 2018)	Regulation of mood, anxiety, and stress response. Essential for the effects of some psychobiotics
Immune	Cytokines (TNF-α, IL-6, IL-1β), LPS	Translocation of microbial components (LPS) across the compromised intestinal barrier, activating systemic immunity. Cytokines circulate and cross/signal across the BBB (Ghosh et al., 2020)	Induction of “sickness behavior,” neuroinflammation, microglia activation, and altered neurotransmitter metabolism
Endocrine	Cortisol, serotonin (5-HT), GABA, dopamine	Modulation of the HPA axis. Intestinal production of neurotransmitters or their precursors that may have local and systemic effects (Wadan et al., 2025)	Response to stress, regulation of mood and anxiety. Over 90% of the body’s serotonin is produced in the gut
Metabolic	Short-chain fatty acids (SCFAs: butyrate, propionate, acetate)	Produced by fiber fermentation. They cross the blood-brain barrier, act as histone deacetylase inhibitors (HDACs), and binders to G protein-coupled receptors (GPCRs) (Duan et al., 2023)	Neuroprotection, microglia maturation, maintenance of BBB integrity, systemic anti-inflammatory effects

The vagus nerve is the most direct and rapid communication pathway. With approximately 80%–90% of its fibers being afferent (from the gut to the brain), it transmits information about the state of the intestinal lumen, including the presence of microbial metabolites and cytokines, directly to brainstem nuclei, such as the nucleus of the solitary tract (NTS) (Bonaz et al., 2018). The integrity of the vagus nerve is crucial for the anxiolytic and antidepressant effects of some probiotics, highlighting its indispensable role in mediating mental health benefits (Bonaz et al., 2018).

SCFAs, primarily butyrate, propionate, and acetate, are the main products of fiber fermentation by the microbiota (Clapp et al., 2017). Butyrate is the main energy source for colonocytes and is essential for maintaining the integrity of the intestinal barrier. Systemically, SCFAs exert anti-inflammatory effects, for example, by promoting the differentiation of Treg cells. They can cross the blood-brain barrier (BBB) and directly influence brain function, modulating the maturation and activity of microglia and exerting neuroprotective effects (Wadan et al., 2025).

Beyond SCFAs, several other classes of microbially-derived metabolites exert profound effects on the gut-brain-immune triad. Bile acids (BAs), synthesized from cholesterol and modified by gut bacteria (e.g., *Bacteroides*), have emerged as essential emotional regulators (Stanimirov et al., 2025). These molecules activate specific receptors such as the Farnesoid X Receptor (FXR) and the G protein-coupled receptor TGR5, both of which are expressed on brain vascular epithelial cells and neurons (Idahosa et al., 2025). Activation of TGR5 in the brain reduces neuroinflammation by inhibiting pro-inflammatory cytokines and enhancing BDNF-mediated synaptic resilience, whereas disturbances in BA metabolism in IBD—characterized by a reduction in secondary BAs—are linked to increased anxiety risk and visceral hypersensitivity (Zhao et al., 2025). Emerging 2025 evidence underscores the role of secondary bile acids (e.g., lithocholic acid, LCA) as critical messengers in the gut–immune–brain axis, where their reduction in IBD patients is directly linked to serotonergic deficits in the prefrontal cortex (Idahosa et al., 2025).

Furthermore, the microbiota is a critical factory for B-complex vitamins and Vitamin K, which are essential for neuronal bioenergetics and blood-brain barrier maintenance. The malabsorptive environment of IBD often leads to deficiencies in these microbially-synthesized vitamins, contributing to cognitive slowing and mood dysregulation (Wang et al., 2025). Finally, the gut microbiota significantly influences the metabolism of xenobiotics, including pharmacological agents and environmental toxins. A healthy microbial community can detoxify potentially neurotoxic substances, while dysbiosis may lead to the production of metabolites that cross the BBB and “prime” microglia, exacerbating neuroinflammatory responses (Sabahat et al., 2024; Yassin et al., 2025). Together, these metabolites form a complex chemical signaling network that dictates the host’s neuroimmunological status.

The microbiota can synthesize or modulate the production of a wide range of neuroactive molecules. Notably, more than 90% of the body’s serotonin (5-HT) is produced by enterochromaffin cells in the gut, a process strongly influenced by microbiota. Bacteria of the genera *Lactobacillus* and *Bifidobacterium* can produce gamma-aminobutyric acid (GABA), the main inhibitory neurotransmitter of the CNS. Signals from the gut also powerfully modulate the hypothalamic-pituitary-adrenal (HPA) axis, the body’s central stress response system (Appleton, 2018). Studies in germ-free mice show they have an exaggerated HPA stress response, which can be normalized by colonization, indicating the microbiota is essential for HPA calibration (Evrensel and Ceylan, 2015; Huang et al., 2016).

In a healthy state, the intestinal barrier maintains a strict separation between the lumen and systemic circulation. However, when this barrier is compromised, microbial components like lipopolysaccharide (LPS)—a component of the outer membrane of Gram-negative bacteria—can translocate into the bloodstream, a state known as metabolic endotoxemia. Circulating LPS potently activates innate immunity throughout the body, triggering the release of pro-inflammatory cytokines that can, in turn, communicate with the brain (Ghosh et al., 2020) (Table 1).

**FIGURE 2:**
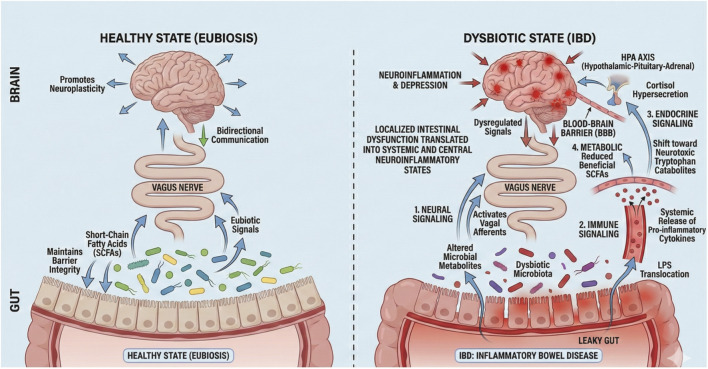
Summary of the complex bidirectional communication within the microbiota-gut-brain axis (MGBA). In a healthy state (eubiosis), the microbiota maintains barrier integrity through the production of short-chain fatty acids (SCFAs), while eubiotic signals via the vagus nerve promote neuroplasticity. However, the dysbiotic state of IBD disrupts this equilibrium through four primary mechanisms: (1) Neural Signaling, where altered microbial metabolites directly activate the vagal afferents; (2) Immune Signaling, involving the translocation of lipopolysaccharides (LPS) across a “leaky gut” and the systemic release of pro-inflammatory cytokines that signal across the blood-brain barrier (BBB); (3) Endocrine Signaling, where microbial dysregulation triggers hyperactivity of the hypothalamic-pituitary-adrenal (HPA) axis and cortisol hypersecretion; and (4) Metabolic Signaling, characterized by a reduction in beneficial SCFAs and a shift toward neurotoxic tryptophan catabolites. This visualized framework demonstrates how localized intestinal dysfunction is translated into the systemic and central neuroinflammatory states characteristic of depression in IBD.

Dysbiosis is an imbalance in the composition and/or function of the intestinal microbial community. It is typically characterized by a reduction in alpha diversity (species richness), a decrease in beneficial butyrate-producing bacteria (e.g., *Faecalibacterium*, *Roseburia*, *Lachnospiraceae*), and an increase in potentially pro-inflammatory bacteria or pathobionts (e.g., *Proteobacteria* like *Escherichia*) (Acevedo-Román et al., 2024; Clapp et al., 2017; Patel et al., 2025). This dysbiotic state has direct pathological consequences. The reduction of butyrate-producing bacteria compromises colonocyte health and intestinal barrier integrity. This, combined with the proliferation of bacteria that can degrade the protective mucus layer, leads to an increase in intestinal permeability, colloquially known as “leaky gut” (Balaprabha et al., 2024; Bosco et al., 2023). A leaky gut allows the translocation of LPS and other microbe-associated molecular patterns (MAMPs) from the lumen into the systemic circulation. Once in the bloodstream, LPS binds to its receptor, Toll-like receptor 4 (TLR4), on immune cells like macrophages, triggering an inflammatory cascade and the systemic production of TNF-α, IL-6, and IL-1β (Duan et al., 2023; Ghosh et al., 2020) (Figure 2).

Although all MGBA pathways are important, in pathological states like IBD, the immune pathway assumes a dominant role. The systemic inflammation generated by dysbiosis and leaky gut not only has direct effects on the brain but also potentiates the other communication pathways. Cytokines can directly activate the vagus nerve, further dysregulate the HPA axis, and alter neurotransmitter metabolism, creating a pathogenic vicious cycle. Thus, the immune pathway acts as a critical amplifier that transforms localized intestinal dysfunction into a systemic disorder with neuropsychiatric manifestations.

## 3 Immunopathogenesis of IBD and its connection to depression

IBD is the archetype of a disease driven by a dysregulated interaction between host genetic susceptibility, environmental factors, and the gut microbiota (Craig et al., 2022; Di Petrillo et al., 2025). The loss of immune tolerance to commensal microorganisms triggers a chronic and destructive inflammatory response in the intestinal mucosa, which serves as the primary source for systemic inflammation that, in turn, affects the brain. The pathogenesis of IBD involves the aberrant activation of both innate and adaptive immunity. Antigen-presenting cells detect microbial antigens that cross a defective epithelial barrier. In IBD patients, these cells activate and promote the differentiation of T helper (Th) cells into pro-inflammatory phenotypes.

In Crohn’s Disease, the immune response is predominantly mediated by Th1 cells (producing IFN-γ and TNF-α) and Th17 cells (secreting IL-17 and IL-22). In Ulcerative Colitis, the response is typically dominated by an atypical Th2 profile, but Th17 activation is also prominent. IL-23 is crucial for maintaining pathogenic Th17 cells, making the IL-23/Th17 pathway a central therapeutic target (Kashani and Schwartz, 2019). These cytokines orchestrate a local “inflammatory storm,” perpetuating tissue damage. Key cytokines like TNF-α, IL-6, IL-1β, and IL-23 are central not only to IBD pathology but are also the same cytokines implicated in the pathophysiology of depression.

A pillar of IBD immunopathogenesis is the dysfunction of the intestinal epithelial barrier. Chronic inflammation directly damages the tight junctions between epithelial cells, increasing intestinal permeability (Lee and Giuliani, 2019). This “leaky gut” allows the massive translocation of microbial components from the lumen into the systemic circulation (Craig et al., 2022). The most potent component is LPS, which serves as a powerful trigger for innate immunity (Craig et al., 2022).

Circulating LPS is recognized by the TLR4 receptor complex, expressed on immune cells throughout the body (Ciesielska et al., 2020). TLR4 activation triggers intracellular signaling pathways, such as the nuclear factor kappa B (NF-κB) pathway (Lu et al., 2008). This culminates in the transcription and release of a cascade of pro-inflammatory cytokines (TNF-α, IL-6, IL-1β), establishing a state of chronic systemic inflammation. This systemic inflammatory state generated in the gut does not remain confined to the periphery; it actively communicates with the CNS, inducing a state of neuroinflammation. Among the multiple mechanisms, the disruption of the blood-brain barrier (BBB), neural and humoral signaling pathways, and microglial activation stand out.

The BBB is a highly selective barrier that protects the brain. Circulating pro-inflammatory cytokines, like TNF-α and IL-1β, can directly increase BBB permeability by decreasing the expression of tight junction proteins (e.g., occludin, claudins) between cerebral endothelial cells (Lee and Giuliani, 2019; Uzzan and Azab, 2021). A compromised BBB allows the entry of cytokines, peripheral immune cells, and other inflammatory molecules into the brain parenchyma. Even without massive disruption, cytokines can signal to the brain. They can be actively transported across the BBB, or they can activate endothelial cells to produce secondary inflammatory mediators (like prostaglandins) that act on the brain. Furthermore, cytokines can activate the afferent fibers of the vagus nerve, which transmit the inflammatory signal directly to the brainstem.

Regardless of the pathway, the final result in the CNS is the activation of microglia, the brain’s resident immune cells. In response to peripheral inflammatory signals, microglia transition from a resting (homeostatic) state to an activated, pro-inflammatory phenotype (da Fonseca et al., 2014). Activated microglia become a primary source of cytokine production (TNF-α, IL-1β, IL-6), chemokines, nitric oxide, and reactive oxygen species *within* the brain itself, establishing and perpetuating the state of neuroinflammation (Fakhfouri et al., 2024). This microglial activation may create a lasting immune memory in the brain, which could explain why depressive symptoms often persist even when intestinal inflammation is in remission (Table 2).

**TABLE 2:** Key inflammatory mediators in the pathogenesis of IBD and comorbid depression.

Mediator	Primary role in the pathogenesis of IBD	Primary role in neuroinflammation and depression
TNF-α	It drives apoptosis of epithelial cells, recruits immune cells, promotes tissue destruction and chronic inflammation	It increases the permeability of the blood-brain barrier, activates microglia, inhibits neurogenesis, and diverts tryptophan metabolism via IDO1
IL-6	It promotes the differentiation of Th17 cells, stimulates the production of acute phase reactants (e.g., CRP), and contributes to systemic inflammation	It crosses the blood-brain barrier, activates microglia, and stimulates hyperactivity of the HPA axis, which is associated with the severity of depressive symptoms
IL-1β	A potent pro-inflammatory cytokine that amplifies the immune response, induces fever, and contributes to tissue damage	It induces “disease behavior,” activates microglia, impairs synaptic plasticity and memory
IL-23	Essential for the survival and expansion of pathogenic Th17 cells, perpetuating intestinal inflammation	It promotes the differentiation of Th17 cells that can infiltrate the CNS, contributing to neuroinflammation
IFN-γ	A key cytokine in the Th1 response, it activates macrophages and increases the expression of MHC molecules, driving inflammation	A major inducer of the IDO1 enzyme, leading to serotonin depletion and the production of neurotoxic kynurenine metabolites
Th17 cells	They produce IL-17 and IL-22, which recruit neutrophils, compromise barrier function, and drive chronic inflammation	Th17 cells can cross the blood-brain barrier and promote inflammation in the central nervous system, and are implicated in models of depression
Microglia	Not applicable (resident cells of the CNS)	Activated by peripheral inflammatory signals, they become the main source of neurotoxic cytokines in the brain, perpetuating neuroinflammation
LPS/TLR4	The translocation of LPS across the damaged intestinal barrier activates TLR4 in immune cells, driving systemic inflammation	Systemic LPS can directly activate microglia and endothelial cells of the BBB, triggering neuroinflammation

The immunoneurobiological connection is further reinforced by the “reciprocal pharmacology” of traditional antidepressants. Beyond their monoaminergic effects, several antidepressants exhibit potent immunomodulatory properties that can directly influence IBD pathology. Paroxetine, a selective serotonin reuptake inhibitor (SSRI), has been identified as a potent inhibitor of G protein-coupled receptor kinase 2 (GRK2), an enzyme often overexpressed in inflammatory states (Ning et al., 2023; Wang et al., 2023). Preclinical models of IBD demonstrate that paroxetine administration significantly alleviates intestinal inflammation by downregulating a broad cascade of pro-inflammatory markers, including TNF-α, IL-1β, IL-6, IL-17a, and IL-23a, while simultaneously reducing the expression of anti-TNF-α-resistance biomarkers like *Osm* and *Osmr* (Tang et al., 2015; Tian et al., 2016).

Mechanistically, paroxetine modulates the structure and function of the gut microbiota, increasing alpha diversity and restoring critical barrier integrity markers such as Mucin 2 (Muc2) and the stem cell marker Lgr5 (Ning et al., 2023; Pinzi et al., 2025). Similarly, SSRIs like fluoxetine and escitalopram have been shown to reduce circulating levels of IL-1β and CRP in patients with Major Depressive Disorder, suggesting that their clinical efficacy may be partially mediated by dampening the systemic “cytokine storm” (Osimo et al., 2019; Tang et al., 2015; Wang et al., 2023). This dual-action profile—where a single agent treats both behavioral symptoms and their underlying immunological drivers—supports the existence of a shared molecular subtype in patients with comorbid IBD and depression, highlighting the potential for integrated pharmacological strategies (Bleibel et al., 2025).

Neuroinflammation driven by microglia activation has devastating consequences for neurochemistry and neuronal plasticity, directly linking immunity to psychiatry. The Kynurenine Pathway (KP) is the primary route for the catabolism of the essential amino acid tryptophan (TRP), accounting for more than 95% of its degradation (Wang et al., 2025). Under physiological conditions, TRP is the precursor for 5-HT synthesis, a neurotransmitter central to mood and cognitive regulation. The KP is considered the “most critical” link between IBD and depression because it serves as a molecular switch that diverts TRP away from the serotonin pathway toward the production of neuroactive metabolites in response to immune activation (Chen et al., 2021). The pathway is initiated by the rate-limiting enzymes indoleamine 2,3-dioxygenase (IDO1/2) and tryptophan 2,3-dioxygenase (TDO). While TDO handles basal metabolism in the liver, IDO is ubiquitously expressed and is potently induced by pro-inflammatory cytokines—specifically interferon-gamma (IFN-γ) and TNF-α—which are hallmarks of active IBD (Dantzer, 2017).

In the context of intestinal inflammation, IDO activation creates a “tryptophan shunt,” leading to: (1) Serotonin Depletion, which directly contributes to the monoaminergic deficit characteristic of MDD; and (2) Neurotoxic Accumulation. Once kynurenine (KYN) crosses the BBB, it is metabolized differently by glial cells. Astrocytes produce the neuroprotective kynurenic acid (KYNA), an NMDA receptor antagonist. However, activated microglia preferentially use the enzyme kynurenine 3-monooxygenase (KMO) to produce 3-hydroxykynurenine (3-HK) and, finally, quinolinic acid (QUIN) (Arnone et al., 2018). QUIN is a potent NMDA receptor agonist that induces excitotoxicity and oxidative stress, leading to the atrophy of hippocampal neurons (Chen et al., 2021). The resulting shift in the KYNA/QUIN ratio—the neuroprotective index—provides a robust mechanistic explanation for how peripheral inflammation is translated into the neurochemical and neuroplastic deficits of depression (Kearns, 2024; Kruse et al., 2019).

Neuroinflammation and neurotoxic KP metabolites like QUIN suppress neurogenesis in the adult hippocampus and reduce levels of Brain-Derived Neurotrophic Factor (BDNF), a molecule essential for neuronal survival, growth, and plasticity (Craig et al., 2022). Low levels of BDNF are one of the most robust and replicated biological findings in MDD. Chronic inflammation and cytokines (especially IL-6 and IL-1β) continuously stimulate the HPA axis, leading to cortisol hypersecretion (Di Petrillo et al., 2025). Over time, this induces a state of glucocorticoid resistance, where receptors become less sensitive to cortisol’s negative feedback. The result is a vicious cycle of uninhibited inflammation and stress response, exacerbating both IBD pathology and depression (Craig et al., 2022).

## 4 Clinical evidence of the association between IBD and depression

The mechanistic link between intestinal inflammation and brain dysfunction is strongly corroborated by a robust body of clinical and epidemiological evidence. Large-scale epidemiological studies and meta-analyses consistently demonstrate that IBD patients have a significantly higher risk of developing anxiety and depression compared to the general population (Barberio et al., 2021; Martin-Subero et al., 2016). A comprehensive meta-analysis found a pooled prevalence of depression symptoms of 25.2% and anxiety symptoms of 32.1% in individuals with IBD (Barberio et al., 2021). These figures represent an approximately two-to three-fold increase relative to healthy controls. According to the 2025 Global Burden of Disease report, MDD remains a leading cause of global disability, with the highest growth rates observed in multimorbid populations in sub-Saharan Africa, where HIV/AIDS and IBD interact to elevate depression risk by 1.9 times (DeLong et al., 2025). The prevalence is even more pronounced during periods of disease activity. In patients with active IBD, the rate of depression symptoms can rise to 35%–40%, and anxiety symptoms can affect 50%–80% of individuals. This suggests a strong correlation between the intensity of peripheral inflammation and the severity of neuropsychiatric symptoms.

The relationship between IBD and depression is not unidirectional. Instead, evidence points to a mutually reinforcing cycle, where intestinal disease can lead to depression, and depression, in turn, can influence the course of IBD (Hinnant et al., 2025). An IBD diagnosis is a strong predictor for the future development of depression. A meta-analysis found an approximately 55% increased risk (Hazard Ratio 1.55) for developing depression after an IBD diagnosis. This pathway is driven not only by the psychosocial burden of living with a chronic disease but also by the biological mechanisms of systemic inflammation and neuroinflammation previously detailed. More intriguingly, a prior diagnosis of depression significantly increases the risk of developing IBD later in life. Meta-analyses show that individuals with a history of depression have a 20%–100% higher risk (HR ranging from 1.2 to 2.0) of subsequently being diagnosed with CD or UC. This finding strongly refutes the simplistic model that depression in IBD is just a psychological reaction. Instead, it strongly suggests the existence of shared biological pathways. Depression is associated with low-grade inflammation, dysbiosis, and HPA axis dysregulation—the same risk factors implicated in IBD pathogenesis. Furthermore, comorbid depression is an independent predictor of a worse IBD prognosis, including higher relapse rates, greater need for hospitalization, and a weaker response to therapies (Di Petrillo et al., 2025; Hinnant et al., 2025).

The identification of objective biomarkers that can predict depression risk in IBD patients is an area of intense research. Classical inflammatory markers, such as CRP and fecal calprotectin, are strongly correlated with intestinal disease activity, which in turn is associated with a higher prevalence of depressive symptoms (Han and Ham, 2021). Elevated CRP levels can even predict which patients with depression (without IBD) will respond better to anti-inflammatory therapies. However, in IBD patients in clinical remission, markers like CRP often do not correlate with the persistence of symptoms like fatigue and depressed mood (Osimo et al., 2019). This “decoupling” suggests that neuroinflammation may become an autonomous process or that symptoms are maintained by lasting neuroplastic changes (e.g., “primed” microglia). This underscores the need for biomarkers that more directly reflect the CNS state. Future research is focused on more sophisticated biomarkers, such as the kynurenine/tryptophan ratio and complex protein panels that may predict comorbid depression with high accuracy.

Recent epidemiological data reveal a significant global shift in the burden of IBD and its psychiatric comorbidities. While IBD prevalence remains highest in Western industrialized nations, incidence rates are rising exponentially in low- and middle-income countries (LMICs) in Asia, Africa, and South America, mirroring the “Westernization” of the gut microbiota (Wang et al., 2026). A 2025 global meta-analysis involving over 150,000 person-years of follow-up demonstrates that IBD patients have a pooled prevalence of depression symptoms of 22.8%–25.2%, and anxiety symptoms of 32.1%–33.8% (Heydari et al., 2025). These regional data emphasize that the gut-brain axis comorbidity is a universal feature of IBD. In South Asia and sub-Saharan Africa, where IBD cases are projected to rise dramatically by 2025, the compounding effect of chronic inflammation and psychological distress creates a critical public health challenge (Barberio et al., 2021; Heydari et al., 2025) (Table 3).

**TABLE 3:** Regional prevalence of depression and anxiety in IBD (2021–2025 data).

Region	Depression prevalence (%)	Anxiety prevalence (%)	Key statistics/Risk factors	Data source
Global pooled	22.8%–25.2%	32.1%–33.8%	2-3x risk vs. general population; risk highest in 1st year post-diagnosis	Luo et al. (2025)
Europe	23.5%	31.0%	Risk Ratio (RR) = 2.0; associated with CRP >3 mg/L	Heydari et al. (2025)
North America	27.2%	13.4%–35.0%	Associated with higher healthcare costs and lost productivity	Abreu et al. (2020)
Asia (East)	15.7%–22.0%	35.7%	Converging with global averages; higher in UC than CD in some cohorts	Qian et al. (2025)
Africa	7.1%–19.6%	5.1%–46.7%	Rapid rise in prevalence; associated with moderate-to-severe disease activity	DeLong et al. (2025)

## 5 Therapeutic implications

The understanding of IBD as a model of inflammatory depression opens new and promising therapeutic avenues. Interventions can be directed at the various nodes of the gut-brain axis pathology: the immune response, the microbiome, and central neurochemical pathways. The evidence that treatments for IBD can alleviate depressive symptoms provides strong support for the inflammatory hypothesis and points toward a more holistic treatment approach. Several drugs can reduce the manifestations of IBD, among them corticosteroids, aminosalicylates, antibiotics and immunosuppressive therapies can be mentioned (Seyedian et al., 2019). The choice for each drug is frequently associated with disease severity and must be made according to the needs and peculiarities of each patient, with surgical resections also being an option in some cases (Seyedian et al., 2019). For patients who are refractory to commonly used drugs, modern therapies such as anti-TNF and different anti-interleukins can also be administered (Balderramo, 2022; Seyedian et al., 2019). In this regard, the main prescriptions for these patients include monoclonal antibodies against TNF - infliximab, adalimumab, golimumab -, IL-12/IL-23 – ustekinumab - and cell adhesion molecules (α4β7) – vedolizumab (Stallmach et al., 2023).

Moreover, immunobiological drugs represent important options for patients with severe disease and present proven efficacy, however their effects in individuals’ mental health still must be analyzed, considering that the anxiety and depression incidences in IBD patients is significantly elevated (Gray et al., 2018; Stallmach et al., 2023). In this sense, the use of TNF-α inhibitors has been linked to an amelioration of self-esteem, wellbeing, sensory functions and alteration of brain responses in left amygdala, right prefrontal cortex, posterior cingulate cortex and primary visual regions, demonstrating modifications in cognitive-affective processing (Gray et al., 2018). According to studies, the alteration of self-perception is frequent in depression, along with the reductions of the speed of cognitive bias, with that in mind, these drugs have shown potential to reduce manifestations of depression in IBD patients (Gray et al., 2018).

In this regard, research also demonstrates that the use of anti-TNF-α drugs presents superior impacts in depressive scenarios when compared to other immunomodulators (Horst et al., 2015). In this sense, studies suggest that both pharmacological classes can reduce depressive symptoms in ulcerative colitis patients, however, the magnitude of the anti-depressant effect of anti-TNF-α drugs was significantly higher, reducing the levels of patients at risk for moderate to severe depression (Horst et al., 2015). Still, Crohn’s disease patients presented significant reduction of depressive symptoms only when using TNF- α inhibitors, not other immunomodulators (Horst et al., 2015).

Nonetheless, a few rare cases of psychiatric adverse effects of anti-TNF-α are present in literature. In this sense, Shayowitz et al. (2019) have presented a case of a 16-year-old IBD patient who presented important depressive symptoms and had a suicide attempt a few days after the infusion of infliximab (Shayowitz et al., 2019). Additionally, another case report associated the infusion of the same drug to panic attacks and the suicide of a 30-year-old IBD patient (Roblin et al., 2006). According to this case, the drug was administered 5 times, and around 2 h after each of the infusions this patient presented severe panic attacks, demonstrating some association of the drug with anxiety symptoms and suicidal behavior in rare cases, highlighting the need for a mental status evaluation before the initiation of the therapy and frequent follow-up by mental health professionals (Roblin et al., 2006). Additionally, these manifestations prove the influence of this class of drugs in central nervous system behavior.

On the other hand, another immunobiological drug, Ustekinumab, an anti-IL12/IL23 drug, has demonstrated important effects in mental health of IBD patients as well. Apparently, the drug seems to reduce fatigue, improve work productivity and health-related quality of life, which comprises depression and anxiety conditions and social and family interactions (Panés et al., 2023). In this sense, fatigue represents one of the main extraintestinal manifestations of IBD, being associated with depressive behavior and impacting sociability and other life spheres, hence the amelioration of this parameter can have important impacts in mental health and other habits, as seen in this study (Panés et al., 2023). Therefore, it also represents a class of immunobiologicals capable of affecting psychological conditions in patients with IBD.

Finally, Vedolizumab, an anti-α4β7 drug, has also presented effects in sleep quality and mood of IBD patients (Stevens et al., 2017). In a cohort study, sleep disturbance was significantly reduced after the beginning of the treatment with this immunobiologic, compared to baseline. Additionally, anxiety and depression scores were also filled by participants in the beginning of the study and after the initiation of the therapy, presenting a reduction of psychological symptoms during the treatment (Stevens et al., 2017). Still, it is important to mention the influence of sleep quality in psychological scenarios and the modification of sleep habits as a manifestation of mental disorders, indicating that the improvement of sleep quality by itself can indicate an improvement in depression and anxiety, as well as have positive impact in mental conditions (Stevens et al., 2017). Hence, the treatment with this drug has also presented an important positive effect on individuals’ psychological conditions.

Regarding the effects of immunobiologics in mental health as a therapy in other autoimmune diseases, similar effects have been seen. As an example, the use of infliximab as a treatment for patients with ankylosing spondylitis has demonstrated positive impacts, reducing depressive and anxious symptoms (Ertenli et al., 2012). Additionally, a study developed by Davies et al. (2020) has demonstrated a similar result. Patients with inflammatory arthritis received anti-TNF-α and presented improvement in depressive symptoms both 24 h after infusion and 12 weeks after the infusion, demonstrating an important impact in mental health. Furthermore, in this research is also hypothesized that the administration of these drugs decreases right amygdala reactivity, explaining the results described by scientists (Davies et al., 2020).

Moreover, the use of adalimumab in psoriasis patients has presented important improvements in quality-of-life parameters as well. According to studies, mental and physical wellbeing, cutaneous body image, anxiety, depression and disease severity conditions were evaluated in a prospective multicenter observational study, presenting an amelioration of all conditions 16 weeks after the beginning of the treatment (Leman et al., 2020). However, the mental status ameliorations were seen simultaneously to the reductions in disease manifestations, indicating that the psychological effects could be also linked to a response to the reduction of dermatological symptomatology (Leman et al., 2020).

Another study, evaluating the effects of immunobiological drugs in moderate-to-severe psoriasis patients has presented similar results. In this study, Ríos-Navarro et al. (2015) have evaluated the effects of both anti-TNF- α and anti-IL12/23 drugs - adalimumab, etanercept and ustekinumab, - finding that patients who received one of the drugs presented improvement in depressive symptoms 12 weeks after the beginning of the treatment, when compared to placebo. Therefore, different immunobiologics have presented similar effects in mental health amelioration (Ríos-Navarro et al., 2015).

Still, it is important to evaluate the bidirectional relationship between disease severity and the need for immunobiological drugs, and the prevalence of depressive symptoms and response to treatment. Studies suggest that patients with severe autoimmune diseases, who are initiating immunobiologics, tend to have a more important impairment of mental health, with a large prevalence of depression than those with lighter manifestations (Hider et al., 2009). Considering that, depressive manifestations were also associated with a worse response to therapies, including immunobiologics, hence indicating a bidirectional influence among the treatment and mental status (Hider et al., 2009). Hence, considering the multifactorial aspects of psychological conditions, it is safe to say that immunobiological drugs can affect mental health through several paths, as well as the diseases themselves can affect responses to these treatments.

With that in mind, to understand the main hypothesized via responsible for the antidepressant effects of immunobiologics it is essential to understand the role of neuroinflammation in depressive disorders. Studies have shown that peripheral inflammatory biomarkers are elevated in patients with depression, including compounds such as TNF-α and interleukins (Nettis and Pariante, 2020). From that, researchers suggest that either the proinflammatory cytokines in peripheral regions might be able to find a breach in blood-brain-barrier and reach the brain, or that those molecules can connect to structures of the peripheral nervous system, such as the nerves, and through them, trigger brain responses (Nettis and Pariante, 2020). Regarding that, patients with inflammatory diseases usually present elevated levels of proinflammatory molecules in the organism, what, along with the burden of the diseases, would explain the higher prevalence of depressive disorders in these groups, as seen in IBD (Uzzan and Azab, 2021).

In this scenario, research indicates that glial cells might be enrolled in the neuroinflammatory processes implied in depressive pathophysiology. According to studies, these cells are affected by the peripheral inflammation, what causes an imbalance in the native immune cells of the central nervous system (Yang et al., 2025). From that point on, the activation of microglia leads to the release of more cytokines, reduction of serotonin levels and production of oxidative stress molecules, explaining the development of depressive symptoms (Nettis and Pariante, 2020). Furthermore, studies also suggest that exposure to stress, both in children and adults, can trigger the proinflammatory response from peripheral immune cells, activating the cascade enrolled in depression pathophysiology and causing damage to the hippocampus, a region where new neurons are produced (Nettis and Pariante, 2020; Wu et al., 2021). Moreover, individual characteristics, such as resilience under stressful conditions, can influence the levels of immune response and the impact in hippocampal neurogenesis, reducing brain damage and depressive responses (Wu et al., 2021).

Furthermore, research suggests that neuroinflammation can also cause an imbalance in the hypothalamic-pituitary-adrenal (HPA) axis. According to studies, this axis is activated in stressful contexts, however, under chronic stress or high intensity stress, this via is impaired, leading to the hypersecretion of cortisol and a reduction in the ability to reduce the secretion of the hormone in peaceful conditions as well (Ehlert et al., 2001). In this scenario, the elevation of cortisol has already been linked to depressive disorders, indicating an association between the imbalance of the axis, neuroinflammation and depression (Ehlert et al., 2001).

Considering that, studies have indicated that the anti-inflammatory characteristics of immunobiological drugs, especially anti-TNF-α, are responsible for a reduction of proinflammatory conditions of IBD patients, reducing neuroinflammation and improving mental status of individuals in this group (Siebenhüner et al., 2021). Still, several antidepressants have been demonstrating anti-inflammatory potential, such as lithium and fluoxetine, indicating that immunobiologics might act through the same path influencing behavior (Uzzan and Azab, 2021). Finally, selective cyclooxygenase-2 inhibitors anti-inflammatories were tested as adjuvant therapies for patients with mood disorders, presenting positive responses, corroborating the neuroinflammatory hypothesis and serving as a preview and explanation to what can be seen regarding the intervention with immunobiologics and the potential of these drugs as antidepressants.

Additionally, it must be highlighted that the effectiveness of these drugs and reduction of IBD manifestations, along with improvement of everyday tasks execution, sociability and sleep quality can also reduce depressive behavior, having a positive impact on individuals’ mental health (Stevens et al., 2017). Hence, it is safe to say that both the anti-inflammatory activities of immunological drugs and the reduction of IBD manifestations can be associated with a better mental condition, especially when considering the multifactorial characteristics of psychological diseases, being impossible to isolate the beneficial responses to one only cause.

However, it is still crucial to develop more studies evaluating the effects of immunobiological drugs in depressive conditions in IBD patients and the mobilized cascades, aiming at understanding the mechanisms enrolled in these processes and how to develop strategies that improve the mental health of those patients. Still, it is important to evaluate the cases where adverse effects impacted negatively on mental health as well, not only to understand more about the activities of these drugs, but to allow a prediction of vulnerabilities than can reduce the safety of prescription of the drugs, collaborating with the security and wellbeing of patients.

Given the central role of dysbiosis, therapies aimed at restoring a healthy microbial ecosystem (eubiosis) are a logical and low-risk therapeutic strategy. Probiotics are live microorganisms that, when administered in adequate amounts, confer a health benefit to the host. Studies in populations with primary depression and irritable bowel syndrome (IBS) have shown that certain strains of *Lactobacillus* and *Bifidobacterium* can alleviate depressive symptoms (Evrensel and Ceylan, 2015; Huang et al., 2016; Miller and Raison, 2016). Proposed mechanisms include improving intestinal barrier function, producing SCFAs, and modulating the immune system, resulting in a systemic anti-inflammatory effect. Prebiotics (fibers that selectively feed beneficial bacteria) and symbiotics (combinations of pre- and probiotics) are also being explored.

Fecal Microbiota Transplantation (FMT) is the transfer of a complete microbial community from a healthy donor to a recipient, representing the most potent form of microbiome manipulation. While highly effective for recurrent *Clostridioides difficile* infection, its application in IBD and depression is still experimental. Preclinical studies are compelling: FMT from healthy donors can reverse depressive-like behaviors in rodents, while FMT from depressed patients can induce these behaviors in healthy animals (Dziedzic et al., 2024; Evrensel and Ceylan, 2015). Small clinical trials and case reports in MDD patients have shown promising improvements in depressive and gastrointestinal symptoms, but large-scale trials are needed (Arneth, 2025).

Diet is one of the most potent modulators of the gut microbiota and inflammation. Western-style diets, rich in saturated fats, refined sugars, and processed foods, promote dysbiosis and inflammation. In contrast, anti-inflammatory diets, such as the Mediterranean diet, rich in fiber, polyphenols, and omega-3 fatty acids, are associated with greater microbial diversity, lower levels of inflammatory markers, and a reduced risk of depression. Specific diets, like the Anti-Inflammatory Diet for IBD (IBD-AID), have been developed to restore microbial balance.

The field of microbiome modulation for mental health has led to the emergence of the term “psychobiotic”. A psychobiotic is defined as a live organism (i.e., a probiotic) that, when ingested in adequate amounts, produces a health benefit in patients suffering from psychiatric illnesses (Heidarzadeh-Rad et al., 2020; Messaoudi et al., 2011; Zhu et al., 2025). This concept represents a targeted evolution of probiotics, focusing on strains with specific neuroactive mechanisms, such as the ability to produce neurotransmitters (e.g., GABA, serotonin), modulate the HPA axis, or exert potent immunomodulatory effects (Akkasheh et al., 2016; Bosco et al., 2023; Ehlert et al., 2001; Siebenhüner et al., 2021). Strains like *Lactobacillus helveticus* R0052 and *Bifidobacterium longum* R0175 have been shown in clinical trials to significantly reduce symptoms of depression and anxiety compared to placebo (Heidarzadeh-Rad et al., 2020; Messaoudi et al., 2011). The identification and validation of effective psychobiotics for IBD-associated depression is an exciting frontier in psychopharmacology and gastroenterology (Benton et al., 2007; Kazemi et al., 2019; Mohammadi et al., 2016) (Table 4).

**TABLE 4:** Summary of key clinical trials on microbiome-targeted therapies for depression.

Autor, year	Intervention (strain/Type)	Population	Duration	Primary psychiatric outcome	Key changes in biomarkers (if measured)
Akkasheh et al. (2016)	L. acidophilus, L. casei, B. bifidum	Patients with MDD	8 weeks	Significant reduction in beck depression Inventory (BDI) score vs. placebo	Decreased CRP, increased glutathione
Messaoudi et al. (2011)	*L. helveticus* R0052, B. longum R0175	Healthy volunteers with stress	30 days	Significant reduction in psychological stress and depression scores versus placebo	Reduction of urinary cortisol
Kazemi et al. (2019)	B. longum R0175, *L. helveticus* R0052	Patients with MDD (adjunctive therapy)	8 weeks	Significant improvement in depression symptoms, possibly due to increased BDNF levels	Significant increase in serum BDNF levels
Mohammadi et al. (2016)	Probiotic yogurt or multi-species capsule	Workers in the petrochemical industry	6 weeks	Beneficial effects on mental health (GHQ and DASS scores) vs. placebo	Not measured
Benton et al. (2007)	Yogurt with *Lactobacillus* casei	Healthy volunteers	3 weeks	Improved mood in individuals with initially poor mood	Not measured

### 5.1 Critical appraisal: associative vs. interventional evidence

In evaluating the gut–immune–brain axis, it is imperative to distinguish between associative findings—which predominate in the current literature—and robust interventional evidence. While a vast body of evidence demonstrates a correlation between elevated pro-inflammatory cytokines (e.g., IL-6, TNF-α) and depressive severity in IBD patients, the causal impact of pharmacological neutralization of these mediators is still being delineated through rigorous clinical trials (Luo et al., 2025). For instance, *post hoc* analyses of the STARDUST trial for ustekinumab and GEMINI trials for vedolizumab suggest significant improvements in fatigue and mood-related quality of life; however, these remain secondary outcomes (Panés et al., 2023).

Furthermore, the field must address neutral or inconsistent outcomes, particularly in microbiome-targeted interventions. While psychobiotics show promise, several high-quality meta-analyses indicate that their efficacy is often most pronounced in mild-to-moderate depression rather than in chronic, treatment-resistant cases where neuroplastic changes may have become independent of gut-derived inflammatory signals (Dacaya et al., 2025). For example, a 2021 meta-analysis involving 705 participants noted that while probiotics generally alleviate MDD symptoms (SMD = −0.292), the causation between microbiota changes and antidepressant clinical response was not uniformly confirmed across all psychometric scales (Misera et al., 2021). This suggests that “one-size-fits-all” probiotic approaches may be less effective than targeted, strain-specific interventions tailored to individual inflammatory profiles (Dacaya et al., 2025; Misera et al., 2021).

### 5.2 Paradoxical psychiatric effects and safety considerations

A critical pharmacological consideration in the use of biological therapies is the emergence of paradoxical psychiatric adverse events (PAEs). While anti-TNF-α agents generally exhibit antidepressant properties by reducing neuroinflammation, a rare but severe spectrum of psychiatric risks has been documented. Large-scale observational studies indicate that infliximab (IFX) exposure is associated with an elevated risk of hospital admission for psychiatric events, with a reported hazard ratio (HR) of 4.5 for psychiatric disorders overall (Thillard et al., 2020). Specifically, the risk of manic episodes is notably higher in patients treated for severe psoriasis (HR = 12.6), and psychotic disorders have been observed with a higher frequency in ulcerative colitis patients treated with IFX (HR = 5.43) (Thillard et al., 2020).

Most alarming are the reports of suicide attempts and depressive destabilization immediately following biological infusion. Case reports have detailed suicide attempts in adolescents and adults with IBD shortly after infliximab administration, necessitating rigorous mental status monitoring during the initiation phase (Shayowitz et al., 2019). These paradoxical effects may stem from a complex cytokine imbalance; blocking TNF-α can lead to a compensatory increase in the production of interferon-alpha (IFN-α) by plasmacytoid dendritic cells, a cytokine well-known for inducing profound “sickness behavior” and clinical depression (Thillard et al., 2020). Thus, while biological therapies are essential for mucosal healing, their impact on the CNS requires careful clinical oversight to manage these rare but life-threatening psychiatric outcomes (Aşkın et al., 2017).

### 5.3 Immunomodulatory potential of conventional antidepressants

Traditional antidepressants exhibit significant “off-target” immunomodulatory properties that contribute to their efficacy in the gut–immune–brain axis. Selective serotonin reuptake inhibitors (SSRIs), such as fluoxetine, escitalopram, and sertraline, have been shown to significantly reduce circulating levels of pro-inflammatory cytokines (IL-1β, IL-2, IL-6, and TNF-α) in depressed patients (Bleibel et al., 2025; Pinzi et al., 2025). Mechanistically, these drugs can inhibit the NLRP3 inflammasome and the NF-κB pathway, both of which are central drivers of IBD pathology (Bleibel et al., 2025).

Furthermore, conventional antidepressants may directly influence the gut barrier and microbiome. SSRIs have been shown in preclinical models to modulate the hypothalamic-pituitary-adrenal (HPA) axis stress response and reduce systemic inflammation, while paroxetine has the unique “added benefit” of lowering intestinal inflammation by restoring butyrate-producing bacteria and barrier markers like Muc2 (Luo et al., 2025; Ning et al., 2023). These findings suggest that antidepressants should be considered not merely as symptomatic mental health treatments, but as systemic immunomodulators that can stabilize the gut source of neuroinflammation, potentially reducing the frequency of IBD clinical flares (Di Petrillo et al., 2025).

## 6 Limitations of the literature and future perspectives

Despite significant advances in understanding the gut-brain link in IBD and depression, the field faces several critical limitations that must be addressed to translate mechanistic knowledge into effective clinical interventions. A fundamental barrier is the lack of a clear, quantitative, and clinically relevant definition of “dysbiosis”. The term is often used vaguely to describe an “imbalance,” a definition that lacks scientific rigor. The composition of the gut microbiota varies enormously among healthy individuals, making it difficult to define a single “eubiotic” profile. This ambiguity hinders the development and regulatory approval of microbiome-targeted therapies. The field needs to move from compositional descriptions (who is there) to functional assessments (what are they doing), such as the reduced capacity to produce SCFAs. Many human studies are correlational, showing associations but not establishing cause and effect (Arneth, 2025; Osadchiy et al., 2018). Although animal models provide strong evidence of causality (Dziedzic et al., 2024), more longitudinal human studies are needed to determine whether dysbiosis is a cause or a consequence of the disease.

A glaring gap is the scarcity of robust clinical trials designed to simultaneously assess gastroenterological and neuropsychiatric outcomes as co-primaries. In most IBD therapy trials, mental health data, if collected, are relegated to secondary outcomes. This limits the ability to draw firm conclusions about the true efficacy of treatments on depressive symptoms. Furthermore, the clinical assessment of depression in IBD patients is inherently complex due to the significant overlap between the somatic symptoms of MDD (e.g., fatigue, sleep disturbances, appetite loss) and the extraintestinal manifestations of IBD itself. This overlap can lead to an overestimation of depression prevalence and diagnostic confusion.

To overcome these limitations, more integrated and sophisticated research approaches are needed, including: prospective cohort studies are crucial, integrating multiomics analyses (metagenomics, metabolomics, proteomics) with detailed neuropsychiatric assessments and functional neuroimaging (Han and Ham, 2021; Yassin, Nakhal, et al., 2025). In addition, future more reliably reflect CNS pathology (neuroinflammation, BBB dysfunction) in patients with IBD. This will allow for better patient stratification and monitoring of treatment response. Finally, the field should evolve from generic interventions to precision therapies, including next-generation psychobiotics with specific mechanism of action and personalized dietary interventions based on the individual’s multiomic profile.

### 6.1 Heterogeneity in neuropsychiatric assessment and somatic overlap

A primary hurdle in the meta-analysis of IBD and depression is the lack of standardized assessment tools. Studies utilize a diverse array of instruments, ranging from self-report questionnaires like the Beck Depression Inventory (BDI-II), Patient Health Questionnaire-9 (PHQ-9), and Hospital Anxiety and Depression Scale (HADS) to clinician-administered scales like the Hamilton Depression Rating Scale (HAM-D) (Hu et al., 2021). This heterogeneity significantly impacts the comparability of prevalence data and therapeutic outcomes. For instance, the HADS is often preferred in IBD research because it omits somatic criteria—such as fatigue, sleep disturbances, and weight changes—that are inherently part of the IBD clinical picture (Vaughan et al., 2019). In contrast, the PHQ-9 maps directly to DSM-5 criteria and includes these somatic domains, which may lead to an overestimation of depression prevalence during active IBD flares (Thombs et al., 2014; Vaughan et al., 2019).

The implications for pharmacological trials are profound. Studies relying on the HADS may report lower sensitivity for detecting minor depressive symptoms compared to the BDI-II or PHQ-9 (Hu et al., 2021). Furthermore, reliance on abbreviated tools like the PHQ-2 can be problematic; a 2025 cohort study showed that 5.7% of IBD patients who screened negative on the PHQ-2 were found to have moderate-to-severe depression or suicidal ideation when evaluated with the full PHQ-9 (Thombs et al., 2014). To resolve these discrepancies, future research must move toward validating IBD-specific psychiatric instruments that can effectively distinguish between inflammatory sickness behavior and primary clinical depression, ensuring that pharmacological interventions are targeted appropriately.

The advantage of utilizing molecular biomarkers for patient stratification lies in the ability to identify clinically relevant “inflamed” subgroups of patients who are unlikely to respond to standard monoaminergic antidepressants. Evidence suggests that approximately 39% of MDD cases exhibit a distinct “inflamed phenotype” characterized by hs-CRP >3 mg/L, elevated IL-6, and increased counts of neutrophils and CD4^+^ T cells (Pinzi et al., 2025). Stratifying patients by these markers, as well as by the glial-derived protein S100B (a marker of BBB dysfunction), can predict which patients are most likely to benefit from biological therapies or psychobiotics (Ning et al., 2023; Osimo et al., 2019).

Furthermore, recent transcriptome-wide studies have identified four highly specific candidate biomarkers for IBD-related MDD: HGF (Hepatocyte Growth Factor), SPARC, ADAM12, and MMP8(Hu et al., 2024). These secreted proteins not only distinguish MDD in the IBD context but also contribute to the pathogenesis of mood disorders through direct interactions with inflammatory immune pathways. By utilizing a “D-score” system based on these biomarkers, clinicians can identify at-risk patients during the early stages of intestinal disease, allowing for the initiation of “add-on” neuroprotective interventions before chronic neuroplastic changes occur (Ning et al., 2023).

## 7 Conclusion

The microbiota–gut–brain axis represents a transformative framework for understanding the bidirectional relationship between IBD and Major Depressive Disorder. This review has established that depression in IBD is not merely a psychological comorbid state but a direct biological consequence of a pathological cascade originating in the gut. Dysbiosis and intestinal barrier failure trigger a systemic “cytokine storm” that drives microglial activation and dysregulates the neurotoxic kynurenine pathway, effectively remodeling the central neurochemical environment.

The significance of these findings is clinical and pharmacological: the management of IBD must shift from a compartmentalized approach to a unified, integrated model of care. By combining highly targeted immunotherapies (e.g., biologics) that dampen systemic inflammation with microbiome-targeted psychobiotics and, when indicated, anti-inflammatory antidepressants like paroxetine, clinicians can achieve both mucosal healing and “neuro-remission”.

Adopting this gut–immune–brain therapeutic mandate is essential for precision medicine. As the field moves toward validating objective molecular biotypes—such as the inflamed endotype defined by hs-CRP thresholds and highly accurate protein panels (HGF, MMP8)—personalized neuroimmunological care becomes a reality. This ensures that the millions of patients living at this complex intersection achieve a true state of multi-organ health, where therapeutic interventions address the full spectrum of both enteric and cerebral manifestations.

## Glossary

3-HK: 3-hydroxykynurenine
5-HT: Serotonin
BAs: Bile acids
BBB: Blood-Brain Barrier
BDI: Beck Depression Inventory
BDNF: Brain-Derived Neurotrophic Factor
CD: Crohn’s Disease
CNS: Central Nervous System
CRP: C-Reactive Protein
DASS: Depression Anxiety Stress Scales
FMT: Fecal Microbiota Transplantation
GABA: Gamma-aminobutyric Acid
GALT: Gut-Associated Lymphoid Tissue
GHQ: General Health Questionnaire
GRK2: G protein-coupled receptor kinase 2
HADS: Hospital Anxiety and Depression Scale
HAM-D: Hamilton Depression Rating Scale
HPA: Hypothalamic-Pituitary-Adrenal Axis
HR: Hazard Ratio
IBD: Inflammatory Bowel Disease
IBD-AID: Anti-Inflammatory Diet for IBD
IBS: Irritable Bowel Syndrome
IDO: Indoleamine 2,3-dioxygenase
IFN-γ: Interferon-gamma
IL: Interleukin
KP: Kynurenine Pathway
KYN: Kynurenine
KYNA: Kynurenic acid
LPS: Lipopolysaccharide
MAMPs: Microbe-Associated Molecular Patterns
MDD: Major Depressive Disorder
MGBA: Microbiota-Gut-Brain Axis
NF-κB: Nuclear Factor kappa B
NMDA: N-methyl-D-aspartate
NTS: Nucleus of the Solitary Tract
PAEs: Paradoxical psychiatric adverse events
PAMPs: Pathogen-Associated Molecular Patterns
PHQ-9: Patient Health Questionnaire-9
QUIN: Quinolinic Acid
SCFAs: Short-Chain Fatty Acids
SSRI: Selective serotonin reuptake inhibitor
TDO: tryptophan 2,3-dioxygenase
Th: T helper cells
TLR4: Toll-like Receptor 4
TNF-α: Tumor Necrosis Factor-alpha
Treg: Regulatory T cells
TRP: Tryptophan
UC: Ulcerative Colitis
